# The Impact of Covid-19 on Women's Experiences of and Through Football in Buenos Aires

**DOI:** 10.3389/fspor.2020.624055

**Published:** 2021-01-18

**Authors:** Juliana Román Lozano, Mónica Santino, David Wood

**Affiliations:** ^1^La Nuestra Fútbol Feminista, Buenos Aires, Argentina; ^2^The University of Sheffield, Sheffield, United Kingdom

**Keywords:** women, football, feminism, Argentina, La Nuestra Fútbol Feminista, Club Atlético Huracán, coronavirus, COVID-19

## Abstract

This research report explores the impact of Covid-19 on women's football in Buenos Aires. The suspension of all forms of football in Argentina as part of the country's hard lockdown measures threatens to undo significant gains made in women's football in recent years. By focussing on the experiences of key actors in a feminist Civil Society Organization (CSO) and a newly professional women's team, respectively, we examine what the pandemic has meant for women's football and for women football players at different levels of the game. We also consider the potential impact of the current situation on the future of women's football in Argentina, representative of wider social advances for women in the country.

## Introduction: Covid-19 and Football in Argentina

The first case of Covid-19 in Argentina was identified on 3 March 2020, with the first death on 7 March, both involving men in Buenos Aires who had returned from Europe. On 11 March, the same day that the WHO declared a pandemic, President Arturo Fernández announced a 14-day quarantine for anyone arriving from high-risk countries, followed on 19 March by the nationwide introduction of one of the strictest lockdowns in Latin America. This lockdown was in place until 26 April, when restrictions were relaxed in some parts of the country, where infection rates were low. However, the capital remained in lockdown until mid-July, with an initial limited relaxation overturned and redoubled after a spike in cases saw the country pass 100,000 cases and 1,900 deaths on 13 July, with 49.7% of cases in the capital, which is home to 7.2% of the population (Ministry of Health, 2020). Within Buenos Aires, the large informal settlement of Villa 31—home to feminist football CSO La Nuestra Fútbol Feminista (henceforth La Nuestra)—became ‘the epicenter for infection over several weeks’ (Acuña, 2020), accounting for almost 50% of new cases in Buenos Aires during late May (Primera Edición: el Diario de Misiones, 2020). During the strict lockdown, no physical exercise outside the home was allowed, and when there was some relaxation of this in early June, the leader of Buenos Aires local government explicitly banned group activities while also acknowledging the risks to women of exercising alone (Infobae, 2020a). Despite maintaining the world's longest-running lockdown through to November 2020 in many parts of the country, including the capital Buenos Aires, Argentina's Ministry of Health announced on 19 October 2020 that they had passed one million confirmed cases of Covid-19, with 26,716 Covid-related deaths to that date (Ministry of Health, 2020). Just over half of the cases, and of the deaths, had been recorded in the province of Buenos Aires, home to the great majority of the country's women's football teams.

Even before the country entered lockdown, the government had announced the suspension of all categories of football in Argentina from 17 March, with the Argentinian Football Association (AFA) announcing in mid-October a scheduled return to action for women's football on 21 November 2020, three weeks after the restart of the first division of men's football and a month after the return of Argentina's top clubs to the Copa Libertadores. In recognition of the financial impact of these measures, AFA and the Argentinian Players' Association (FAA) reached an agreement to pay the salaries of players between July and December 2020 in cases where contracts expired at the end of June, as well as social security for the players and their families. This was applicable to the top four tiers of the men's leagues and to the first division of the three-tier national women's league (Palazzo, 2020). However, the minimal requirements for the professionalization of women's football, discussed further below, meant that the first two footballers in Argentina to test positive for Covid-19, both of whom live in Villa 31, were not covered by these provisions. Stephanie Rea, the goalkeeper for Club Atlético Excursionistas, was not one of the eight players awarded a contract by the club, and Camila Godoy plays in the reserve team of River Plate, excluded from the provision agreed by the AFA and FAA several weeks after both had tested positive (Infobae, 2020b,c). Their situation is illustrative of the extremely challenging situation, and the lack of various forms of support, facing women football players across the country in the pandemic.

## Materials

### Women and Football in Argentina

Football is by far the most popular sport in Argentina for men and women, both as spectators and—increasingly for women—players. It has been played by women since at least 1923, when the first women's club, Río de la Plata, was founded in Buenos Aires, consisting of three teams, two of which played against each other at the Boca Juniors ground in October that year (Pujol, 2019: 265-74; Elsey and Nadel, 2019: 28-29). During the same decade, football became closely linked to notions of masculinity and national identity and numerous respected studies of football in Argentina over recent decades have focused exclusively on the men's game while overlooking entirely the presence of women's football (Archetti, 1994, 1999; Frydenberg, 2011). In doing so, they have perpetuated and augmented the myth of the national game as a male domain while symbolically excluding female players from arguably the most significant mode of national discourse. Women's football in Argentina has received a degree of institutional support since October 1991, when the AFA started a Women's Football Championship involving eight teams, all from the Province of Buenos Aires. This followed in the wake of the first Women's South American Championship, organized by CONMEBOL earlier that year, and preceded by a month the inaugural Women's World Cup organized by FIFA. Only recently has this history and presence of the women's game in Argentina been recognized (Román Lozano, 2018; Santino, 2018; Wood, 2018; Elsey and Nadel, 2019; Garton, 2019; Pujol, 2019; Saralegui and Rodríguez, 2019), with the profile of women's football greatly enhanced by the strong performance of the national team at the 2019 Women's World Cup in France.

**Figure 1:**
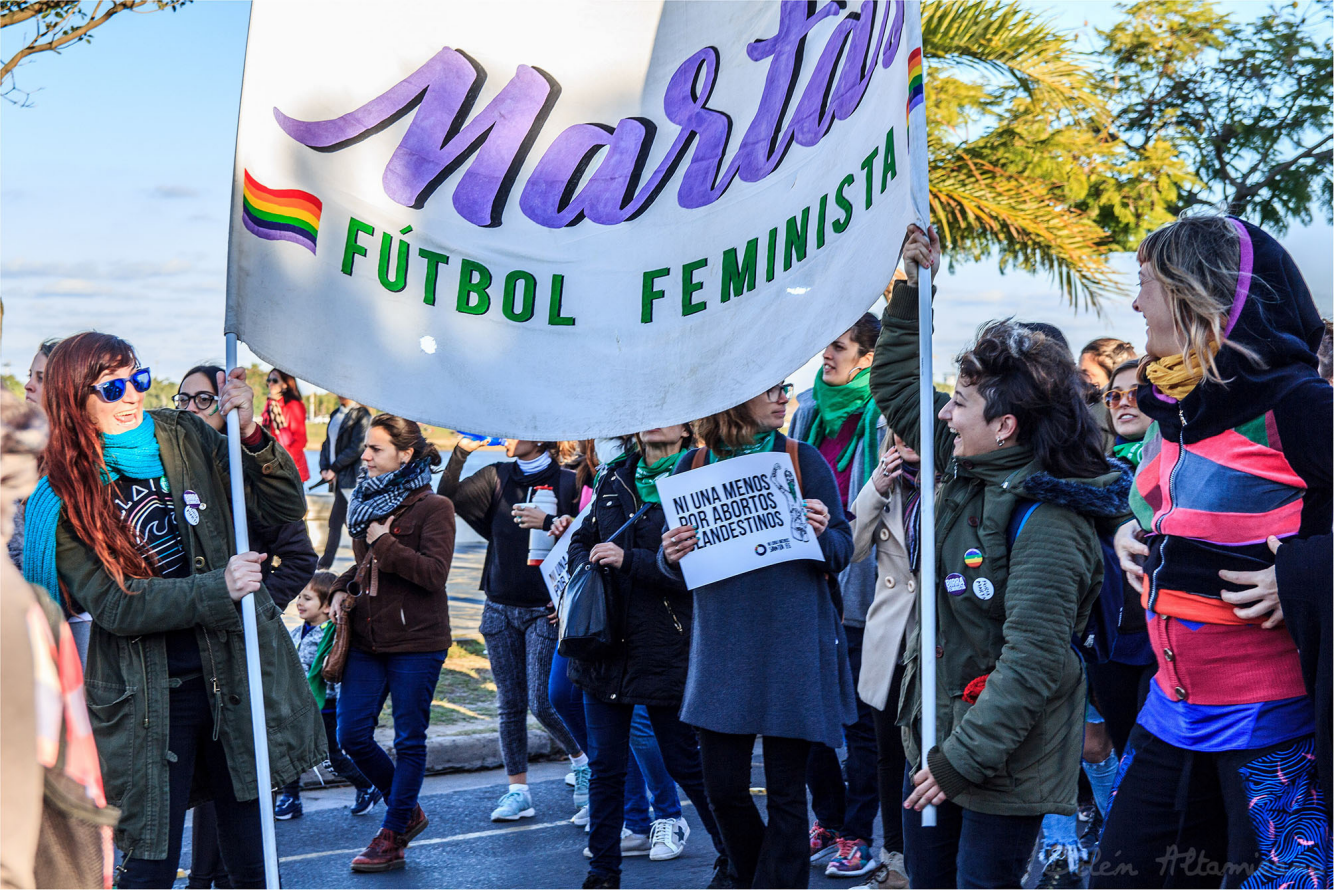
Las Martas Fútbol Feminista at NiUnaMenos rally, Sante Fe, Argentina, 3 June 2018 (Credits: Belén Altamirano, CC-BY-SA-4.0; the image is unchanged from the original, which is available at https://commons.wikimedia.org/wiki/File:Niunamenos_-_3J_-_2018_-_Santa_Fe_-_Argentina0-50.jpg).

The increased visibility of women's football in Argentina has been particularly evident in the capital and has occurred within the framework of powerful grass-roots feminist activism that has drawn on the country's tradition of human rights protests to achieve an impact that extends well beyond Argentina Figure 1. Of particular significance here is the Ni Una Menos movement, whose protest against local femicides and gender violence first came to international prominence through a mass demonstration in central Buenos Aires attended by an estimated 300,000 people on 3rd June 2015, with simultaneous demonstrations in some 80 cities across Argentina and highly effective use of social media since then (Accossatto and Sendra, 2018; Castro Riaño, 2018). From the outset, a wide range of feminist organizations were involved in the movement, including two of the co-authors of this piece, with the support of various other prominent sporting figures, such as Lionel Messi and Juan Martín Del Potro. The movement quickly spread across Latin America and beyond, with the annual Buenos Aires demonstration in 2017 having as a key demand the release of “Higui” De Jesús, a football player with La Nuestra who was imprisoned for defending herself against a gang rape to “correct” her lesbianism (Pujol, 2019: 149-59).

The close connections between feminist activism and football are exemplified by the case of Macarena Sánchez Jeanney, the first woman in Argentina to sign a professional contract as a football player. After taking her club and AFA to court to gain recognition as an employee, having been released halfway through the 2018–19 season with no possibility of joining another team, AFA announced on 16 March 2019 that the national first division of women's football would be made professional. However, clubs would only have to offer contracts to eight players, with salaries pegged to those of players in the men's national fourth division, resulting in semi-professionalism for some players at best. Reflecting on her experience, Sánchez stated that in Argentina “women's football is intrinsically feminist” as “women's movements look to claim realms previously destined exclusively for men” (Sánchez Jeanney, 2019: 222). Prior to the national lockdown triggered by Covid-19, football had become an important means through which girls and women in Argentina, and especially Buenos Aires, accessed physical activity, public spaces and national discourses as football players.

## Methodology: Action Research and Cultural Agency

To respond to the consequences of the Covid-19 lockdown for the women's football clubs that serve as our case studies, and to provide perspectives on the pandemic's impact on women coaches and players, this report will use an essentially qualitative approach that is aligned with action research (AR) and the concept of cultural agency. Our approach to AR is based on an understanding of it as “not so much a *methodology* as an *orientation to inquiry* that seeks to create participative communities of inquiry in which qualities of engagement, curiosity and question posing are brought to bear on significant practical issues” (Reason and Bradbury, 2008: 1). This approach corresponds closely with the practice of La Nuestra as a football community, as discussed below. We furthermore emphasize the nature of AR as context-specific and focussed on a particular problem, on which researchers and participants work together as part of a process of change (Waterman et al., 2001). The co-authors have been collaborating, as Principal Investigator and Project Partners, respectively, on an international research network to explore the practice and representation of women's football, funded by the UK Arts and Humanities Research Council and involving researchers and practitioners from the UK, Argentina, Brazil and Colombia. For our purposes, we draw on the manner in which cultural agency “pursues the tangents of daily practices to multiply creative engagements with power” (Sommer, 2006: 20), understanding power to be present in Argentina in football as a national discourse; in the institutional structures that influence football and the state's response to the Covid-19 pandemic; and in the production of knowledge. In this latter regard especially we recognize cultural agency's value as “a reciprocal dynamic between cultural activists and scholars [that] works productively [to] inspire an original scholarly essay” (Sommer, 2006: 9).

In the presentation of results and discussion below, the use of the first-person plural responds to our emphasis on the collaborative nature of our work as AR (Waterman et al., 2001) and to our “collective sense of intersubjective agreement” (Sommer, 2006: 14) as practitioners of cultural agency. In material below on La Nuestra Fútbol Feminista it also speaks to the positioning of Santino and Román as integral members of that community, activist participants as well as native researchers, with Wood a marginal native able to convey the material effectively from Spanish into English. The emotional investment in the project of Santino and Román in particular informs discussion of the results in relation to La Nuestra and Club Atlético Huracán, respectively, at the same time making explicit the ‘intersubjective agreement’ on which the report is based. In this sense, and to borrow Spivak's important distinction, this essay represents—rather than re-presents—the community to which it relates and from which it speaks (Spivak, 1988).

## Results

### La Nuestra Fútbol Feminista Before Covid-19

**Figure 2:**
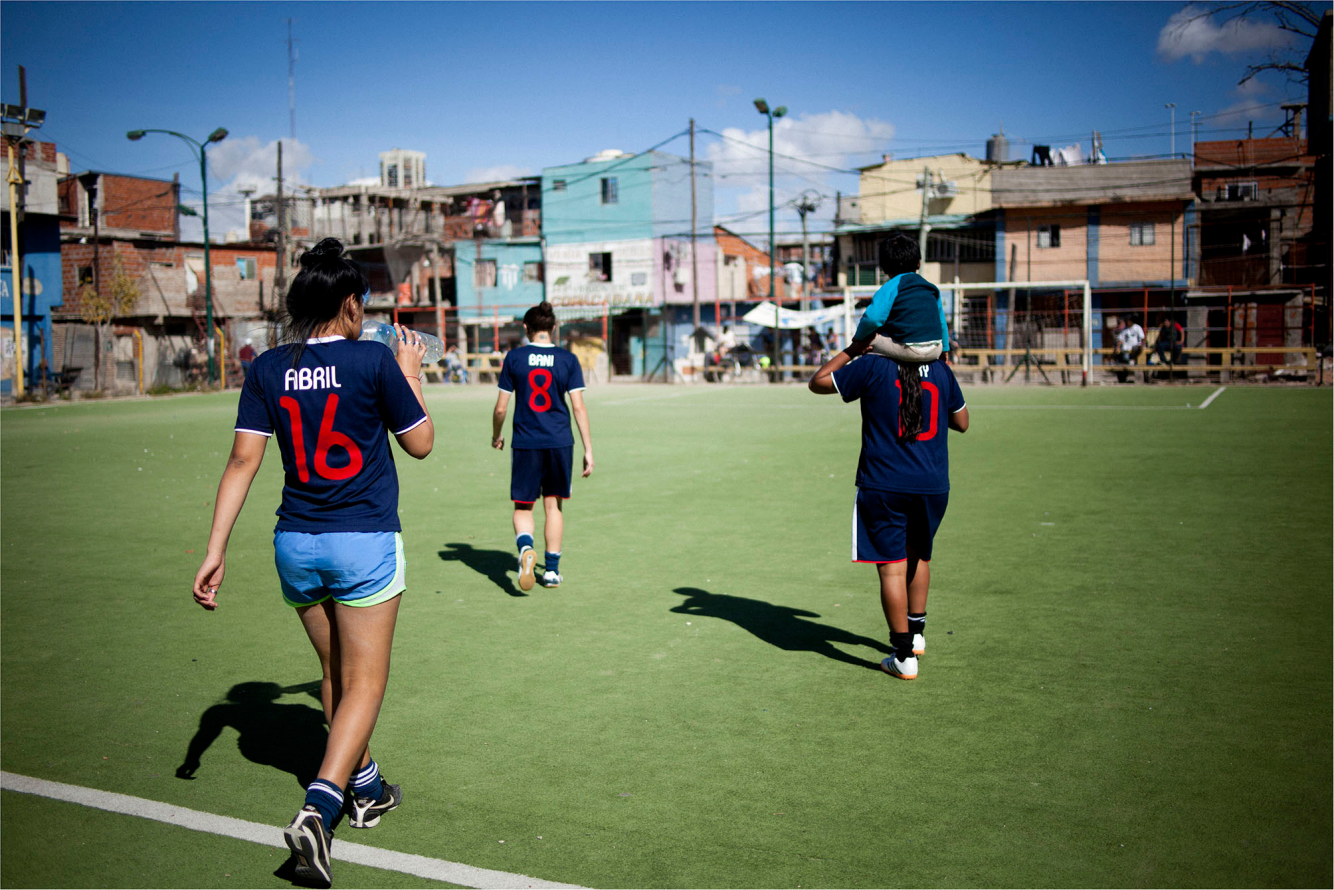
Players taking the pitch used by La Nuestra at Villa 31, Buenos Aires, Argentina (Credits: Sub.Coop; original image available at http://www.sub.coop/es/historias/el-sueno-de-las-pibas-es).

Since 2007, the Civil Society Organization La Nuestra Fútbol Feminista has devoted its praxis to football in the district of Padre Mugica—Villa 31 in Buenos Aires, Argentina. La Nuestra has traditionally been used to refer to a style of play and a performance of national culture with its origins in the 1920s, when local football teams sought to distinguish themselves from the British “fathers” of the game in Argentina (Archetti, 1999). By appropriating the term La Nuestra to name our organization we reject the historic exclusion of women from Argentina's traditional style of play and claim our place as participants in the national game, in which women have also been active for a century. La Nuestra expresses pride in our identity as a group and alludes to possession of the ball and to the fact that playing football is not only about results but is an important opportunity to bring people together. Our activities are aimed at all young and teenage girls, as well as young and older women, opening up the game to any woman, self-identified woman or other non-conformist identity, who wants to play football. Activities are free to all and are underpinned by a coaching staff made up entirely of women. Our practice is based upon a pedagogy of popular education, with a community-based and feminist perspective, rooted in a particular location, and is delivered via the articulation of two days per week of training with a group workshop. Through this dual focus, the sport becomes a space for support, reflection and expression in which the women participate, as well as a space in which to construct our own way of talking about things, to shape an identity that is characteristic of football played by women. In this sense, La Nuestra seeks to guarantee for all participants a space of belonging, from which they can build relationships, challenge the forms of oppression they experience and deploy strategies that allow for the individual and collective empowerment of women, all based on the desire to play football Figure 2.

La Nuestra is conceived, constructed and inhabited by all the players who take part, building in a collective and horizontal fashion a space where they can all exercise their right to play, to free time, to a life without violence. The training sessions and workshops also see the provision of spaces where participants can assume new challenges, such as coordinating skills development sessions, workshops, talks, production of theoretical and audio-visual materials, and coaching. One of the players once said that “I stand tall on the pitch as I do in life,” which we believe offers a synthesis of part of our work, in which participants are not simple recipients, but rather protagonists of the organization: through their suggestions and actions they inhabit and transform the project, turning it into something they consider to be “their place,” “their crowd.”

**Figure 3:**
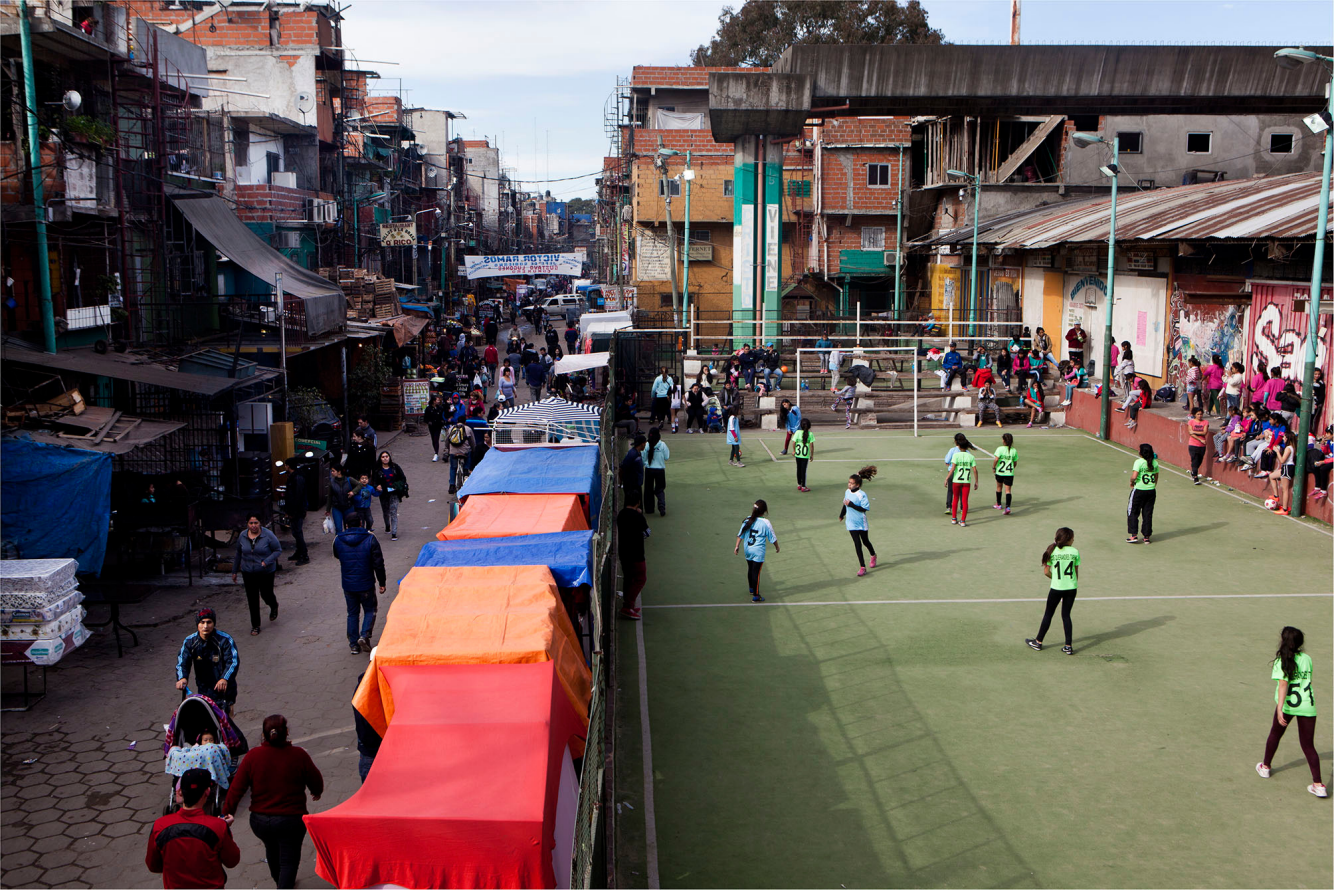
Girls from La Nuestra play on a small pitch adjacent to shops and market stalls in Villa 31 (Credits: Sub.Coop; original image available at http://www.sub.coop/es/historias/el-sueno-de-las-pibas-es).

Over the course of the Organization's 13 years, we can point to several milestones that speak to our collective efforts, to the empowerment of women players and to the achievement of our objectives. Keeping our training sessions going on the most heavily used pitch in the neighborhood, which is also a public space, was the first example of resistance to the oppression we suffered, and to the negation of this space, in order to exercise our right to play. For the last few years, the football pitch in Güemes has been known as “the women's pitch.” As a result of our visibility in the neighborhood, and of our resistant presence on the pitch that we had come to occupy, girls and young women began to demand their own space where they could train. This demand was met by the Organization, and those girls who years ago asked for their rights are now reference points for the youngest girls Figure 3. In 2007, about 12 teenage girls were involved in the project; in 2020 there are more than 120 players of different ages who support La Nuestra's activities, part of—and feeling that they are part of—the project.

At the same time, given the constant process of questioning and reflection that we carry out from our particular perspective when we play football, we came to recognize that our practice entails a deep pool of knowledge and that we needed to build links with other groups and organizations in order to broaden the landscape of women's football. As a result, in recent years we have made a collective effort to develop theory based on our practice, systematizing our shared experiences within the spaces of our workshops. What arose from this rethink was the need to propose new meeting spaces, leading to the promotion of workshops and festivals with other collectives and organizations, something that all the participants in La Nuestra took on as a goal.

### The Impact of Covid-19 on La Nuestra Fútbol Feminista

The current circumstances have highlighted the difficult conditions and profound inequalities that the inhabitants of Padre Mugica district—male and female—face on a daily basis. The lack of access to health provision, to basic services such as water and electricity, and the high levels of unemployment exacerbate historic conditions of marginality and abandonment by the state. The policy of compulsory social isolation because of Covid-19 has proved especially difficult and painful as women and girls often have not only to live with their aggressors but also do so in crowded and precarious conditions. Living together in a family bubble is made all the more difficult for teenage girls and young women, many of whom have been going through a process of individual and collective empowerment by being part of La Nuestra. As a result of the social isolation measures they have been obliged to (re)take traditional gender roles under the new family dynamics that mean more time spent living together and more use of the shared domestic space. Many of the players from La Nuestra have expressed their anxiety and sadness at experiencing this resurgence and reinforcement of those traditional roles and demands that years of struggle on the pitch had helped to unpick. At the same time, the prejudices toward the inhabitants of the informal settlements and popular districts of Buenos Aires have been intensified because of the exponential rise in Covid-19 cases that was evident in mid-2020 as a result of eight days without the provision of any water. Many residents lost their jobs and others were forced to stay at home without being able to go out to seek their daily sustenance.

In the face of this new context, in which the struggle for life and dignity comes first, we have seen our organization called upon to modify its practices and dynamically adapt our modus operandi in response to emerging priorities. While we have organized ourselves around the health crisis facing the district in which we have been working for the last 13 years, we have at the same time been challenged by the profound changes that sports in general are undergoing, and especially sport practiced by women. During this pandemic we have seen how inequality and social injustice have become even more obvious and have deepened, and for our organization the decision to support our players and their families with food and other basic necessities has had a big emotional and political impact. Organizing a campaign for donations to enable the delivery of food, cleaning products and disinfectant reinforced the fabric of new support strategies on behalf of women's networks. Thanks to these alliances that are being created and strengthened, we have been able to maintain this support for 22 weeks and will continue to do so for as long as we are able.

From the sporting perspective, we have adapted our training sessions to the virtual world while bearing in mind the various issues and challenges that the players face, such as lack of internet connectivity, the limited space available to many and the absence of computers or mobile phones that can enable us to meet online. Above and beyond these difficulties, we have adapted so we can stay close to each other, facing up to the pandemic together and standing tall on this new pitch as we stand tall in life.

### Reflections on the Experience of La Nuestra Fútbol Feminista

A social and health crisis exploded in our district in May 2020. The main causes were the lack of drinking water, inadequate living conditions and long-standing structural problems in the informal settlements of Buenos Aires that no government has resolved with effective policies. Against this backdrop, it is women who occupied the front line to confront the crisis, maintaining communal cooking and eating spaces as well as the care of children and the elderly. Many women were also the first to put their lives at risk, with some of them contracting the virus and subsequently dying.

For La Nuestra, this very difficult, unprecedented and complex situation leaves the organization facing new challenges. Our thirteen-year journey in the neighborhood, cemented through close and powerful affective bonds, means that the starting point from which we confront this uncertain panorama is strong. Feminist networks, the power to organize and women's capacity to work are the best evidence of the strengths that this social collective possesses, and these tools will have to serve us on the new paths and scenes that present themselves. Recovering the football pitch, making it once again a territory that is desired, fought for and shared with more women, and gradually reintroducing face-to-face training sessions, will allow us to rebuild with the same conviction that made La Nuestra an organization with such a presence.

Collective endeavor and efforts to exercise the right to play football turned our ideas into fully-fledged convictions that will not be abandoned. We see our sporting practice as a mode of empowerment to eradicate gender violence and in this new setting we will have to redouble our efforts so as not to lose any of the ground gained on the pitch as a space of freedom and in the deconstruction of established prejudices in relation to what is expected of bodies and behaviors.

### The Argentinian Women's First Division/ Club Atlético Huracán Before Covid-19

Following the creation of the Women's Football Championship on 26 October 1991, Buenos Aires-based Club Atlético Huracán took part for the first time in 1999, since when it has remained an ever-present member of the Championship. From the outset, the existence of Huracán's women's team has owed more to the desire, commitment and efforts of certain individuals than to institutional investment and support for women's football as a project. Since 1999 the conditions for training, the quality of the coaching staff (often lacking formal accreditation), the lack of access to club facilities, of travel funds, of medical staff, of adequate kit, among other factors, has been reflected in the team's performance: Huracán has consistently been in the bottom half of the table, with the exception of the 2010/11 season, when they finished in third place.

The process of professionalization of women's football, which began in Argentina in March 2019, has not resulted in significant changes for Huracán in terms of investment or support for the discipline. In these regards, the club fulfilled the minimum requirements stipulated by AFA to be able to compete in what is now Argentina's Professional First Division of Women's Football. The first professional squad of women's football that was registered by Club Atlético Huracán consisted of 32 players, of which eight (the minimum requirement) were awarded the contracts paid for by AFA, with no further investment by the club. As a result, only the eight players who signed professional contracts would have access to medical insurance and to social security provided by the FAA; the remainder of the squad enjoyed no such rights. At the same time, the club maintained the minimum salary (c.$250, equivalent to salaries in the men's fourth division) for the eight players with a contract, with no supplements or increases. Of the seventeen teams that played in the women's professional league in 2019/20 only four had a coaching team led by a woman, and only one—Huracán—a coaching team consisting of two women.

### The Impact of Covid-19 on Club Atlético Huracán and the Women's First Division

As a result of the declaration of a pandemic by the World Health Organization on 11 March 2020, the President of Argentina put in place ‘preventative and obligatory social distancing’ for all the country's inhabitants. Arising from the lockdown that came into force, citizens were prohibited from taking to streets, paths and public spaces and there was also a ban on all types of sporting activities carried out in groups. In the face of this situation the coaching team had to redesign not only the ways in which coaching sessions were carried out, via a virtual platform, but also the ways in which social relations, support and communications with the players were to be conceived.

The process of adaptation was gradual and had various stages of trial and refinement. It is worth pointing out that players' participation in the virtual training sessions was heavily affected by lack of access to a computer, or to a mobile phone with capacity to download the app being used, as well as the lack of access to an internet connection for almost half of the squad. As lockdown progressed it became increasingly obvious that most of the players and the coaching staff were experiencing a sense of profound marginalization, a situation that aggravated the ongoing inequality of structural conditions that the club had developed over the years for the women players and coaching staff.

Most of the players earn their living from informal jobs or live with their family, who assume their living costs and who in turn have no access to stable employment. Because of the lockdown that came with Covid-19, many players lost any work they had and with it their primary source of income. On a related note, since the start of the (semi-)professional women's championship, the income of seven of the eight contracts awarded to Huracán players were shared among all 32 members of the squad, which provided each player with a minimal travel allowance, although this brought with it no employment rights. During the initial period of the lockdown this situation worsened considerably as the club fell behind by three months on salary payments and when eventually payments were made, many players could not access the small amount of shared funds as they were unable to meet up with their teammates because of the ongoing travel restrictions within Buenos Aires.

Over the course of several months, the players and coaching staff endured considerable uncertainty as there was a lack of regular communications from the club and no clear sense of what would happen with players' contracts or continuity of the squad and coaching staff. Following intense negotiations between the FAA and AFA, AFA released a briefing that ensured the extension of players' contracts until December 2020 and also gave the possibility for clubs to extend the contracts of coaching staff until December 2020. With certainty on that front, but no official communication from the club amid ongoing questions, the squad continued to work until 22 June 2020, training from Monday to Friday and holding online meetings twice a week. During the week of 22 June the Head of Women's Football at Club Atlético Huracán told the coaching team via a zoom call that they were unable to renew their contract, although “they assured us that it was simply down to economics for if it was a question of sporting principles the club would have hired a male coaching team from the outset.”

### Reflections on the Experience of Club Atlético Huracán

Club Atlético Huracán is an example of what is happening in many football clubs in Argentina. Women in the realm of football still face deep inequalities that are maintained and reinforced by institutional practices at every level, and which this pandemic has rendered even more visible. The small number of women who have secured a contract as a football player, not only at Huracán but in all teams, face a huge salary gap when compared to their male peers. Most players do not have medical insurance nor access to basic services in order to practice their sport in adequate conditions. The pandemic has reinforced this marginal status of women players in terms of access to healthcare for them and their families. Fewer than five percent of women players in Argentina live solely on their income from football, which is why women footballers have to resort to a double (or even triple) working day or emigrate to be able to develop as players and make a living from the sport. Over the first 6 months of the pandemic, at least 10 Argentinian women footballers, from the best teams in the country, have moved abroad, mostly to Europe, given that local teams were unable to guarantee adequate salaries or a prompt and safe return to training and competitive matches.

For women coaches the outlook is similar or even worse. Both practically and symbolically, football is constructed as a masculine domain, which translates, among other things, into an absence of women in the labor market for roles in football. The salary gap between male and female coaches working in the men's and women's first division is huge, but even so wage cuts and sackings during Covid-19 have fallen primarily on women coaches. The impact of Covid-19, and all the restrictions and changes that it has entailed for football in Argentina, reveals the highly precarious position of women players, women coaches and other women working in the sport in terms of their daily experiences and their working conditions.

While there have been formal changes aimed at creating spaces that are free from discrimination against women in daily life as in sport, these changes are not reflected in women's daily experiences in the various domains of football. At the same time, it is vital to point out that the immediate changes in day-to-day life that the pandemic triggered for women in football (loss of employment, support and basic needs), as well as the key decisions and structural reforms that have been introduced during the first 6 months of lockdown in Argentina, have consistently revealed a patriarchal logic as far as agendas and priorities are concerned. For example, in June 2020 CONMEBOL decided to suspend the clause that required clubs competing in the (men's) Copa Libertadores to provide investment and support for their women's teams. This, and various other examples, reveal how high-level decisions result in backward steps in relation to the attainment of spaces and visibility, as well as to rights that had been secured, all to the detriment of the development and quality of life of women who are footballers, coaches and referees, or who occupy other roles in the field of women's football.

In order to be able to imagine a post-Covid-19 development of football for all, and especially to be able to contemplate the sport as a space free from discrimination and various forms of violence, there is a need for concrete actions within footballing institutions so that more women and a greater diversity of gender perspectives are present in decision-making, in knowledge-sharing and in policy design. Only then will it be possible to talk of real changes in the development of the sport for women.

## Discussion of Results

Following the (limited) professionalization of the women's game in March 2019 and the national team's performances at the Women's World Cup in July 2019, women's football in Argentina had made significant gains in terms of media coverage and institutional support, and these achievements were being reflected in wider social attitudes to it as an important element of the feminist movement. As is evident from the experiences of La Nuestra and Club Atlético Huracán, Covid-19 has had a profound impact on the practice of women's football in Buenos Aires and Argentina more widely, both at the newly professional level as well as for amateur clubs, grass-roots organizations and all of their players.

The pandemic, of course, had an impact on men's football too, but the structural inequalities that were only starting to be addressed in terms of salaries, access to medical provision, equipment and facilities have re-emerged in force, magnifying the impact of the pandemic on women playing for and working in clubs in the nation's top three divisions. In contrast with their male counterparts, only a handful of those women players who receive a salary from their First Division club are able to live exclusively on that income, with clear consequences for living arrangements and the ability to maintain social distancing, resulting in higher risks of infection from the virus. The symbolic capital that women's football was rapidly gaining before the pandemic has been lost for the time being: with women's league football still to resume at the time of writing (November 2020), and the Women's Copa Libertadores postponed indefinitely, men's football has returned to dominate exclusively the media as the group stage of the Copa Libertadores resumed with a flurry of matches from mid-September and the (men's) First Division played its first round of matches at the end of October 2020. Below the women's First Division, where players do not receive salaries, support is limited to AFA's provision of Covid-19 testing and arranging permits so that players can make their way to future matches on public transport.

As the experience of La Nuestra has shown, the impact on women's grass-roots football has been even more acute. In addition to the material challenges of the pandemic in relation to access to basic services such as food, drinking water and healthcare, the many women and girls who play football in the crowded informal settlements of Buenos Aires face considerable challenges to be able to keep fit, train and practice. Of even greater potential significance, however, is the social, emotional, and mental impact of the pandemic and the hard lockdown that it has entailed. Women and girls for whom football provided a space of empowerment to question and challenge traditional structures, roles and bodily practices have been forced to return to the confines of domestic spaces in which traditional gender roles may be retained and reinforced. In a country where 54.2% of men over the age of 14 take no part at all in domestic tasks (Anuario Estadístico de la República Argentina, 2020: 197), many girls and women who found expression on a football pitch find themselves expected to (re)adopt roles as cooks, cleaners, and carers.

At this stage it is impossible to evaluate the long-term impact on the physical, emotional and mental health of women football players in Argentina, but for now it is clear that the pandemic has seen the rolling back of numerous gains made by and for women in recent years, in football as in society more widely. Our hope is that women's football is able to resume its recently secured practical and symbolic position within the country as quickly as it has been lost over recent months: many people involved in women's football will be doing all they can to ensure that this is the case.

## Recommendations for Women's Football in Light of Covid-19

In relation to the findings discussed above, we make a series of recommendations that may serve to address the issues identified therein:

- Extended availability and accessibility of wi-fi services and online platforms to enable the retention and continued development of established communities and networks;
- More effective communication of information in relation to public health measures and support offered by state institutions (medical centers, access to subsidies, reporting gender violence);
- Development of feminist networks and of alliances with other organizations both within and beyond the neighborhood to share lessons learned toward common goals;
- Development of closer relations with the families of women football players in order to provide more effective support for their sporting activities;
- Retain the limited gains made for women's football in national sporting institutions rather than decoupling it from support for the men's game as a response to Covid-19;
- Provision of salary and access to medical insurance for all players in the Argentinean Women's Football Championship;
- Access to facilities for training and matches that is equal to that enjoyed by men's teams;
- Provision of public security measures to protect women from gender violence when practicing football and other sports in public spaces;
- Equal access to all roles within football (e.g. playing, coaching, management, refereeing), according to qualifications and experience, and regardless of gender;
- Media reporting and public discussion of women's football on the same terms as men's football, i.e. not as a sexualised spectacle subject to a gender hierarchy;
- Appointment of women to positions of authority within football and inclusion of a greater diversity of voices and experiences within decision-making and policy forums.

The implementation of these measures would bring about a step change in the experience of women playing football, and of Argentinean women more widely, addressing a series of issues that have been highlighted and exacerbated as a result of the Covid-19 pandemic.

## Data Availability Statement

The original contributions presented in the study are included in the article/supplementary material. Further inquiries can be directed to the corresponding author/s.

## Author Contributions

All authors have made a substantial and direct contribution to the work and approved it for publication.

## Conflict of Interest

The authors declare that the research was conducted in the absence of any commercial or financial relationships that could be construed as a potential conflict of interest.
